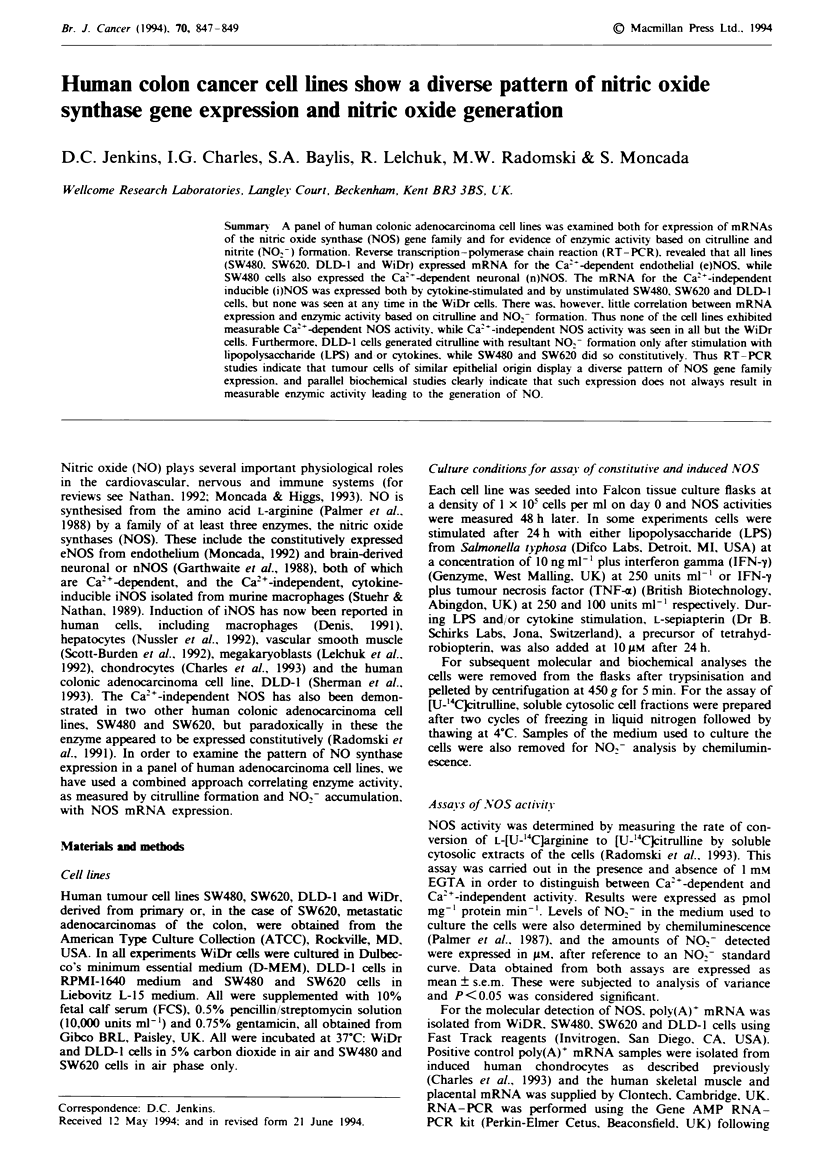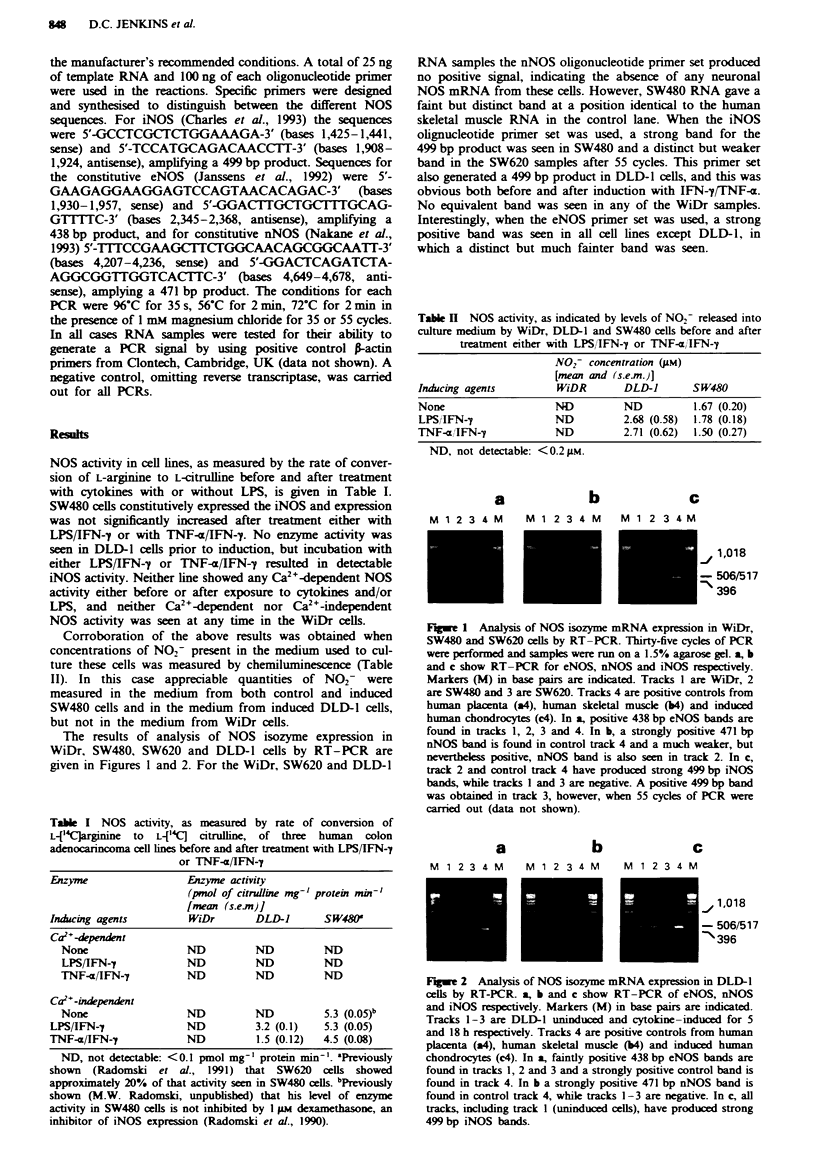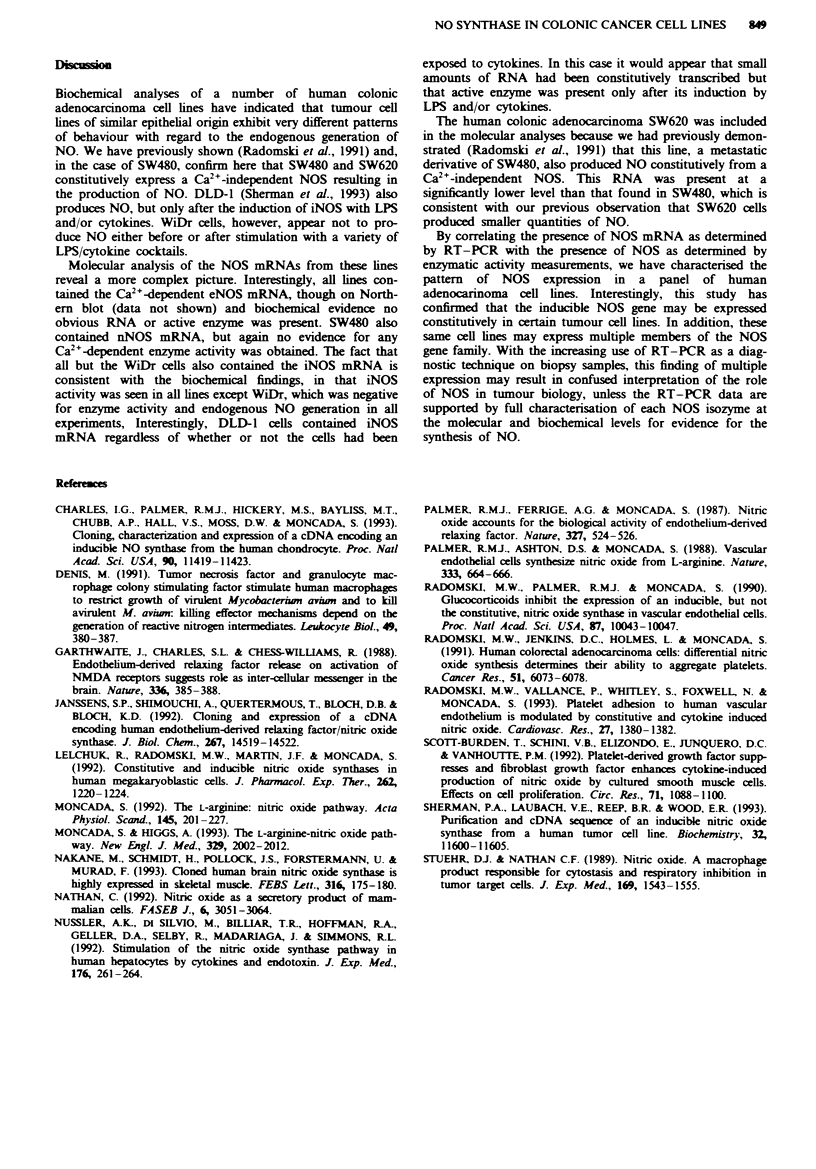# Human colon cancer cell lines show a diverse pattern of nitric oxide synthase gene expression and nitric oxide generation.

**DOI:** 10.1038/bjc.1994.409

**Published:** 1994-11

**Authors:** D. C. Jenkins, I. G. Charles, S. A. Baylis, R. Lelchuk, M. W. Radomski, S. Moncada

**Affiliations:** Wellcome Research Laboratories, Beckenham, Kent, UK.

## Abstract

**Images:**


Br. J. Cancer (1994), 70, 847-849 © Macmillan Press Ltd., 1994

### Human colon cancer cell lines show a diverse pattern of nitric oxide synthase gene expression and nitric oxide generation

D.C. Jenkins, I.G. Charles, S.A. Baylis, R. Lelchuk, M.W. Radomski & S. Moncada

*Wellcome Research Laboratories, Langley Court, Beckenham, Kent BR3 3BS, UK.*

**Summary** A panel of human colonic adenocarcinoma cell lines was examined both for expression of mRNAs of the nitric oxide synthase (NOS) gene family and for evidence of enzymic activity based on citrulline and nitrite ($NO_2^-$) formation. Reverse transcription-polymerase chain reaction (RT-PCR), revealed that all lines (SW480, SW620, DLD-1 and WiDr) expressed mRNA for the $Ca^{2+}$-dependent endothelial (e)NOS, while SW480 cells also expressed the $Ca^{2+}$-dependent neuronal (n)NOS. The mRNA for the $Ca^{2+}$-independent inducible (i)NOS was expressed both by cytokine-stimulated and by unstimulated SW480, SW620 and DLD-1 cells, but none was seen at any time in the WiDr cells. There was, however, little correlation between mRNA expression and enzymic activity based on citrulline and $NO_2^-$ formation. Thus none of the cell lines exhibited measurable $Ca^{2+}$-dependent NOS activity, while $Ca^{2+}$-independent NOS activity was seen in all but the WiDr cells. Furthermore, DLD-1 cells generated citrulline with resultant $NO_2^-$ formation only after stimulation with lipopolysaccharide (LPS) and/or cytokines, while SW480 and SW620 did so constitutively. Thus RT-PCR studies indicate that tumour cells of similar epithelial origin display a diverse pattern of NOS gene family expression, and parallel biochemical studies clearly indicate that such expression does not always result in measurable enzymic activity leading to the generation of NO.

Nitric oxide (NO) plays several important physiological roles in the cardiovascular, nervous and immune systems (for reviews see Nathan, 1992; Moncada & Higgs, 1993). NO is synthesised from the amino acid L-arginine (Palmer *et al.*, 1988) by a family of at least three enzymes, the nitric oxide synthases (NOS). These include the constitutively expressed eNOS from endothelium (Moncada, 1992) and brain-derived neuronal or nNOS (Garthwaite *et al.*, 1988), both of which are $Ca^{2+}$-dependent, and the $Ca^{2+}$-independent, cytokine-inducible iNOS isolated from murine macrophages (Stuehr & Nathan, 1989). Induction of iNOS has now been reported in human cells, including macrophages (Denis, 1991), hepatocytes (Nussler *et al.*, 1992), vascular smooth muscle (Scott-Burden *et al.*, 1992), megakaryoblasts (Lelchuk *et al.*, 1992), chondrocytes (Charles *et al.*, 1993) and the human colonic adenocarcinoma cell line, DLD-1 (Sherman *et al.*, 1993). The $Ca^{2+}$-independent NOS has also been demonstrated in two other human colonic adenocarcinoma cell lines, SW480 and SW620, but paradoxically in these the enzyme appeared to be expressed constitutively (Radomski *et al.*, 1991). In order to examine the pattern of NO synthase expression in a panel of human adenocarcinoma cell lines, we have used a combined approach correlating enzyme activity, as measured by citrulline formation and $NO_2^-$ accumulation, with NOS mRNA expression.

### Materials and methods

### Cell lines

Human tumour cell lines SW480, SW620, DLD-1 and WiDr, derived from primary or, in the case of SW620, metastatic adenocarcinomas of the colon, were obtained from the American Type Culture Collection (ATCC), Rockville, MD, USA. In all experiments WiDr cells were cultured in Dulbecco's minimum essential medium (D-MEM), DLD-1 cells in RPMI-1640 medium and SW480 and SW620 cells in Liebovitz L-15 medium. All were supplemented with 10% fetal calf serum (FCS), 0.5% pencillin/streptomycin solution (10,000 units $ml^{-1}$) and 0.75% gentamicin, all obtained from Gibco BRL, Paisley, UK. All were incubated at 37°C: WiDr and DLD-1 cells in 5% carbon dioxide in air and SW480 and SW620 cells in air phase only.

### Culture conditions for assay of constitutive and induced NOS

Each cell line was seeded into Falcon tissue culture flasks at a density of $1 \times 10^5$ cells per ml on day 0 and NOS activities were measured 48 h later. In some experiments cells were stimulated after 24 h with either lipopolysaccharide (LPS) from *Salmonella typhosa* (Difco Labs, Detroit, MI, USA) at a concentration of 10 ng $ml^{-1}$ plus interferon gamma (IFN-γ) (Genzyme, West Malling, UK) at 250 units $ml^{-1}$ or IFN-γ plus tumour necrosis factor (TNF-α) (British Biotechnology, Abingdon, UK) at 250 and 100 units $ml^{-1}$ respectively. During LPS and/or cytokine stimulation, L-sepiapterin (Dr B. Schirks Labs, Jona, Switzerland), a precursor of tetrahydrobiopterin, was also added at 10 μM after 24 h.

For subsequent molecular and biochemical analyses the cells were removed from the flasks after trypsinisation and pelleted by centrifugation at 450 *g* for 5 min. For the assay of [U-$^{14}$C]citrulline, soluble cytosolic cell fractions were prepared after two cycles of freezing in liquid nitrogen followed by thawing at 4°C. Samples of the medium used to culture the cells were also removed for $NO_2^-$ analysis by chemiluminescence.

### Assays of NOS activity

NOS activity was determined by measuring the rate of conversion of L-[U-$^{14}$C]arginine to [U-$^{14}$C]citrulline by soluble cytosolic extracts of the cells (Radomski *et al.*, 1993). This assay was carried out in the presence and absence of 1 mM EGTA in order to distinguish between $Ca^{2+}$-dependent and $Ca^{2+}$-independent activity. Results were expressed as pmol $mg^{-1}$ protein $min^{-1}$. Levels of $NO_2^-$ in the medium used to culture the cells were also determined by chemiluminescence (Palmer *et al.*, 1987), and the amounts of $NO_2^-$ detected were expressed in μM, after reference to an $NO_2^-$ standard curve. Data obtained from both assays are expressed as mean ± s.e.m. These were subjected to analysis of variance and $P < 0.05$ was considered significant.

For the molecular detection of NOS, poly(A)$^+$ mRNA was isolated from WiDR, SW480, SW620 and DLD-1 cells using Fast Track reagents (Invitrogen, San Diego, CA, USA). Positive control poly(A)$^+$ mRNA samples were isolated from induced human chondrocytes as described previously (Charles *et al.*, 1993) and the human skeletal muscle and placental mRNA was supplied by Clontech, Cambridge, UK. RNA-PCR was performed using the Gene AMP RNA-PCR kit (Perkin-Elmer Cetus, Beaconsfield, UK) following

Correspondence: D.C. Jenkins.
Received 12 May 1994; and in revised form 21 June 1994.

the manufacturer's recommended conditions. A total of 25 ng of template RNA and 100 ng of each oligonucleotide primer were used in the reactions. Specific primers were designed and synthesised to distinguish between the different NOS sequences. For iNOS (Charles *et al.*, 1993) the sequences were 5′-GCCTCGCTCTGGAAAGA-3′ (bases 1,425–1,441, sense) and 5′-TCCATGCAGACAACCTT-3′ (bases 1,908–1,924, antisense), amplifying a 499 bp product. Sequences for the constitutive eNOS (Janssens *et al.*, 1992) were 5′-GAAGAGGAAGGAGTCCAGTAACACAGAC-3′ (bases 1,930–1,957, sense) and 5′-GGACTTGCTGCTTTGCAG-GTTTTC-3′ (bases 2,345–2,368, antisense), amplifying a 438 bp product, and for constitutive nNOS (Nakane *et al.*, 1993) 5′-TTTCCGAAGCTTCTGGCAACAGCGGCAATT-3′ (bases 4,207–4,236, sense) and 5′-GGACTCAGATCTA-AGGCGGTTGGTCACTTC-3′ (bases 4,649–4,678, antisense), amplying a 471 bp product. The conditions for each PCR were 96°C for 35 s, 56°C for 2 min, 72°C for 2 min in the presence of 1 mM magnesium chloride for 35 or 55 cycles. In all cases RNA samples were tested for their ability to generate a PCR signal by using positive control β-actin primers from Clontech, Cambridge, UK (data not shown). A negative control, omitting reverse transcriptase, was carried out for all PCRs.

### Results

NOS activity in cell lines, as measured by the rate of conversion of L-arginine to L-citrulline before and after treatment with cytokines with or without LPS, is given in Table I. SW480 cells constitutively expressed the iNOS and expression was not significantly increased after treatment either with LPS/IFN-γ or with TNF-α/IFN-γ. No enzyme activity was seen in DLD-1 cells prior to induction, but incubation with either LPS/IFN-γ or TNF-α/IFN-γ resulted in detectable iNOS activity. Neither line showed any $Ca^{2+}$-dependent NOS activity either before or after exposure to cytokines and/or LPS, and neither $Ca^{2+}$-dependent nor $Ca^{2+}$-independent NOS activity was seen at any time in the WiDr cells.

Corroboration of the above results was obtained when concentrations of $NO_2^-$ present in the medium used to culture these cells was measured by chemiluminescence (Table II). In this case appreciable quantities of $NO_2^-$ were measured in the medium from both control and induced SW480 cells and in the medium from induced DLD-1 cells, but not in the medium from WiDr cells.

The results of analysis of NOS isozyme expression in WiDr, SW480, SW620 and DLD-1 cells by RT–PCR are given in Figures 1 and 2. For the WiDr, SW620 and DLD-1 RNA samples the nNOS oligonucleotide primer set produced no positive signal, indicating the absence of any neuronal NOS mRNA from these cells. However, SW480 RNA gave a faint but distinct band at a position identical to the human skeletal muscle RNA in the control lane. When the iNOS olignucleotide primer set was used, a strong band for the 499 bp product was seen in SW480 and a distinct but weaker band in the SW620 samples after 55 cycles. This primer set also generated a 499 bp product in DLD-1 cells, and this was obvious both before and after induction with IFN-γ/TNF-α. No equivalent band was seen in any of the WiDr samples. Interestingly, when the eNOS primer set was used, a strong positive band was seen in all cell lines except DLD-1, in which a distinct but much fainter band was seen.

**Table I** NOS activity, as measured by rate of conversion of L-[$^{14}$C]arginine to L-[$^{14}$C] citrulline, of three human colon adenocarincoma cell lines before and after treatment with LPS/IFN-γ or TNF-α/IFN-γ

| *Enzyme* / *Inducing agents* | *Enzyme activity (pmol of citrulline mg$^{-1}$ protein min$^{-1}$ [mean (s.e.m)]* WiDr | DLD-1 | SW480[a] |
|---|---|---|---|
| *$Ca^{2+}$-dependent* | | | |
| None | ND | ND | ND |
| LPS/IFN-γ | ND | ND | ND |
| TNF-α/IFN-γ | ND | ND | ND |
| *$Ca^{2+}$-independent* | | | |
| None | ND | ND | 5.3 (0.05)[b] |
| LPS/IFN-γ | ND | 3.2 (0.1) | 5.3 (0.05) |
| TNF-α/IFN-γ | ND | 1.5 (0.12) | 4.5 (0.08) |

ND, not detectable: <0.1 pmol mg$^{-1}$ protein min$^{-1}$. [a]Previously shown (Radomski *et al.*, 1991) that SW620 cells showed approximately 20% of that activity seen in SW480 cells. [b]Previously shown (M.W. Radomski, unpublished) that his level of enzyme activity in SW480 cells is not inhibited by 1 μM dexamethasone, an inhibitor of iNOS expression (Radomski *et al.*, 1990).

**Table II** NOS activity, as indicated by levels of $NO_2^-$ released into culture medium by WiDr, DLD-1 and SW480 cells before and after treatment either with LPS/IFN-γ or TNF-α/IFN-γ

| *Inducing agents* | *$NO_2^-$ concentration (μM) [mean and (s.e.m.)]* WiDR | DLD-1 | SW480 |
|---|---|---|---|
| None | ND | ND | 1.67 (0.20) |
| LPS/IFN-γ | ND | 2.68 (0.58) | 1.78 (0.18) |
| TNF-α/IFN-γ | ND | 2.71 (0.62) | 1.50 (0.27) |

ND, not detectable: <0.2 μM.

**Figure 1** Analysis of NOS isozyme mRNA expression in WiDr, SW480 and SW620 cells by RT–PCR. Thirty-five cycles of PCR were performed and samples were run on a 1.5% agarose gel. **a**, **b** and **c** show RT–PCR for eNOS, nNOS and iNOS respectively. Markers (M) in base pairs are indicated. Tracks 1 are WiDr, 2 are SW480 and 3 are SW620. Tracks 4 are positive controls from human placenta (**a**4), human skeletal muscle (**b**4) and induced human chondrocytes (**c**4). In **a**, positive 438 bp eNOS bands are found in tracks 1, 2, 3 and 4. In **b**, a strongly positive 471 bp nNOS band is found in control track 4 and a much weaker, but nevertheless positive, nNOS band is also seen in track 2. In **c**, track 2 and control track 4 have produced strong 499 bp iNOS bands, while tracks 1 and 3 are negative. A positive 499 bp band was obtained in track 3, however, when 55 cycles of PCR were carried out (data not shown).

**Figure 2** Analysis of NOS isozyme mRNA expression in DLD-1 cells by RT-PCR. **a**, **b** and **c** show RT–PCR of eNOS, nNOS and iNOS respectively. Markers (M) in base pairs are indicated. Tracks 1–3 are DLD-1 uninduced and cytokine-induced for 5 and 18 h respectively. Tracks 4 are positive controls from human placenta (**a**4), human skeletal muscle (**b**4) and induced human chondrocytes (**c**4). In **a**, faintly positive 438 bp eNOS bands are found in tracks 1, 2 and 3 and a strongly positive control band is found in track 4. In **b** a strongly positive 471 bp nNOS band is found in control track 4, while tracks 1–3 are negative. In **c**, all tracks, including track 1 (uninduced cells), have produced strong 499 bp iNOS bands.

### Discussion

Biochemical analyses of a number of human colonic adenocarcinoma cell lines have indicated that tumour cell lines of similar epithelial origin exhibit very different patterns of behaviour with regard to the endogenous generation of NO. We have previously shown (Radomski *et al.*, 1991) and, in the case of SW480, confirm here that SW480 and SW620 constitutively express a $Ca^{2+}$-independent NOS resulting in the production of NO. DLD-1 (Sherman *et al.*, 1993) also produces NO, but only after the induction of iNOS with LPS and/or cytokines. WiDr cells, however, appear not to produce NO either before or after stimulation with a variety of LPS/cytokine cocktails.

Molecular analysis of the NOS mRNAs from these lines reveal a more complex picture. Interestingly, all lines contained the $Ca^{2+}$-dependent eNOS mRNA, though on Northern blot (data not shown) and biochemical evidence no obvious RNA or active enzyme was present. SW480 also contained nNOS mRNA, but again no evidence for any $Ca^{2+}$-dependent enzyme activity was obtained. The fact that all but the WiDr cells also contained the iNOS mRNA is consistent with the biochemical findings, in that iNOS activity was seen in all lines except WiDr, which was negative for enzyme activity and endogenous NO generation in all experiments, Interestingly, DLD-1 cells contained iNOS mRNA regardless of whether or not the cells had been exposed to cytokines. In this case it would appear that small amounts of RNA had been constitutively transcribed but that active enzyme was present only after its induction by LPS and/or cytokines.

The human colonic adenocarcinoma SW620 was included in the molecular analyses because we had previously demonstrated (Radomski *et al.*, 1991) that this line, a metastatic derivative of SW480, also produced NO constitutively from a $Ca^{2+}$-independent NOS. This RNA was present at a significantly lower level than that found in SW480, which is consistent with our previous observation that SW620 cells produced smaller quantities of NO.

By correlating the presence of NOS mRNA as determined by RT-PCR with the presence of NOS as determined by enzymatic activity measurements, we have characterised the pattern of NOS expression in a panel of human adenocarinoma cell lines. Interestingly, this study has confirmed that the inducible NOS gene may be expressed constitutively in certain tumour cell lines. In addition, these same cell lines may express multiple members of the NOS gene family. With the increasing use of RT-PCR as a diagnostic technique on biopsy samples, this finding of multiple expression may result in confused interpretation of the role of NOS in tumour biology, unless the RT-PCR data are supported by full characterisation of each NOS isozyme at the molecular and biochemical levels for evidence for the synthesis of NO.

### References

CHARLES, I.G., PALMER, R.M.J., HICKERY, M.S., BAYLISS, M.T., CHUBB, A.P., HALL, V.S., MOSS, D.W. & MONCADA, S. (1993). Cloning, characterization and expression of a cDNA encoding an inducible NO synthase from the human chondrocyte. *Proc. Natl Acad. Sci. USA*, **90**, 11419-11423.

DENIS, M. (1991). Tumor necrosis factor and granulocyte macrophage colony stimulating factor stimulate human macrophages to restrict growth of virulent *Mycobacterium avium* and to kill avirulent *M. avium*: killing effector mechanisms depend on the generation of reactive nitrogen intermediates. *Leukocyte Biol.*, **49**, 380-387.

GARTHWAITE, J., CHARLES, S.L. & CHESS-WILLIAMS, R. (1988). Endothelium-derived relaxing factor release on activation of NMDA receptors suggests role as inter-cellular messenger in the brain. *Nature*, **336**, 385-388.

JANSSENS, S.P., SHIMOUCHI, A., QUERTERMOUS, T., BLOCH, D.B. & BLOCH, K.D. (1992). Cloning and expression of a cDNA encoding human endothelium-derived relaxing factor/nitric oxide synthase. *J. Biol. Chem.*, **267**, 14519-14522.

LELCHUK, R., RADOMSKI, M.W., MARTIN, J.F. & MONCADA, S. (1992). Constitutive and inducible nitric oxide synthases in human megakaryoblastic cells. *J. Pharmacol. Exp. Ther.*, **262**, 1220-1224.

MONCADA, S. (1992). The L-arginine: nitric oxide pathway. *Acta Physiol. Scand.*, **145**, 201-227.

MONCADA, S. & HIGGS, A. (1993). The L-arginine-nitric oxide pathway. *New Engl. J. Med.*, **329**, 2002-2012.

NAKANE, M., SCHMIDT, H., POLLOCK, J.S., FORSTERMANN, U. & MURAD, F. (1993). Cloned human brain nitric oxide synthase is highly expressed in skeletal muscle. *FEBS Lett.*, **316**, 175-180.

NATHAN, C. (1992). Nitric oxide as a secretory product of mammalian cells. *FASEB J.*, **6**, 3051-3064.

NUSSLER, A.K., DI SILVIO, M., BILLIAR, T.R., HOFFMAN, R.A., GELLER, D.A., SELBY, R., MADARIAGA, J. & SIMMONS, R.L. (1992). Stimulation of the nitric oxide synthase pathway in human hepatocytes by cytokines and endotoxin. *J. Exp. Med.*, **176**, 261-264.

PALMER, R.M.J., FERRIGE, A.G. & MONCADA, S. (1987). Nitric oxide accounts for the biological activity of endothelium-derived relaxing factor. *Nature*, **327**, 524-526.

PALMER, R.M.J., ASHTON, D.S. & MONCADA, S. (1988). Vascular endothelial cells synthesize nitric oxide from L-arginine. *Nature*, **333**, 664-666.

RADOMSKI, M.W., PALMER, R.M.J. & MONCADA, S. (1990). Glucocorticoids inhibit the expression of an inducible, but not the constitutive, nitric oxide synthase in vascular endothelial cells. *Proc. Natl Acad. Sci. USA*, **87**, 10043-10047.

RADOMSKI, M.W., JENKINS, D.C., HOLMES, L. & MONCADA, S. (1991). Human colorectal adenocarcinoma cells: differential nitric oxide synthesis determines their ability to aggregate platelets. *Cancer Res.*, **51**, 6073-6078.

RADOMSKI, M.W., VALLANCE, P., WHITLEY, S., FOXWELL, N. & MONCADA, S. (1993). Platelet adhesion to human vascular endothelium is modulated by constitutive and cytokine induced nitric oxide. *Cardiovasc. Res.*, **27**, 1380-1382.

SCOTT-BURDEN, T., SCHINI, V.B., ELIZONDO, E., JUNQUERO, D.C. & VANHOUTTE, P.M. (1992). Platelet-derived growth factor suppresses and fibroblast growth factor enhances cytokine-induced production of nitric oxide by cultured smooth muscle cells. Effects on cell proliferation. *Circ. Res.*, **71**, 1088-1100.

SHERMAN, P.A., LAUBACH, V.E., REEP, B.R. & WOOD, E.R. (1993). Purification and cDNA sequence of an inducible nitric oxide synthase from a human tumor cell line. *Biochemistry*, **32**, 11600-11605.

STUEHR, D.J. & NATHAN C.F. (1989). Nitric oxide. A macrophage product responsible for cytostasis and respiratory inhibition in tumor target cells. *J. Exp. Med.*, **169**, 1543-1555.